# Comparison of The Effectiveness of Clomiphene Citrate versus Letrozole in Mild IVF in Poor Prognosis Subfertile Women with Failed IVF Cycles 

**DOI:** 10.22074/ijfs.2015.4542

**Published:** 2015-10-31

**Authors:** Mesut Oktem, Ismail Guler, Mehmet Erdem, Ahmet Erdem, Nuray Bozkurt, Onur Karabacak

**Affiliations:** Department of Obstetrics and Gynecology, Gazi University School of Medicine, Ankara, Turkey

**Keywords:** ICSI, Ovarian Response, Clomiphene Citrate, Letrozole, Ovarian Stimulation

## Abstract

**Background:**

Our objective was to evaluate the effectiveness of clomiphene citrate (CC) vs. letrozole (L) plus human menopausal gonadotropin (hMG) in gonadotropin releasing hormone (GnRH) antagonist protocol in poor prognosis women with previous failed ovarian stimulation undergoing intracytoplasmic sperm injection (ICSI).

**Materials and Methods:**

This retrospective cohort study included cycles with CC and L plus hMG/GnRH antagonist protocols of 32 poor responders who had failed to have ideal follicles to be retrieved during oocyte pick-up (OPU) or embryo transfer (ET) at least for 2 previous *in vitro* fertilization (IVF) cycles with microdose flare protocol or GnRH antagonist protocol from January 2006 to December 2009. Main outcome measures were implantation, clinical pregnancy and live birth rates per cycle. Duration of stimulation, mean gonadotropin dose used, endometrial thickness, number of mature follicles, serum estradiol (E_2_) and progesterone (P) levels on the day of human chorionic gonadotropin (hCG) administration, number of retrieved oocytes and fertilization rates were also evaluated.

**Results:**

A total number of 42 cycles of 32 severe poor responders were evaluated. Total gonadotropin consumption was significantly lower (1491 ± 873 vs. 2808 ± 1581 IU, P=0.005) and mean E_2_ level on the day of hCG injection were significantly higher in CC group than L group (443.3 ± 255.2 vs. 255.4 ± 285.2 pg/mL, P=0.03). ET, overall pregnancy and live birth rates per cycle were significantly higher in CC than L protocol (27.2 vs. 15%, 13.6 vs. 0% and 4.5 vs. 0%, respectively, P<0.05).

**Conclusion:**

Severe poor responders who had previously failed to respond to microdose or GnRH antagonist protocols may benefit from CC plus hMG/GnRH antagonist protocol despite high cancellation rate.

## Introduction

A poor responder has been defined as an infertile woman that develops ≤3 follicles after controlled ovarian hyperstimulation with conventional stimulation protocols in *in vitro* fertilization ( IVF ) ( ESHRE consensus ). The management of poor responders with a history of recurrent failure in conventional microdose protocol or antagonist IVF cycles is difficult and controversial. Recurrent poor response is associated with high financial costs and emotional distress in these couples. There is still no sufficient data and standard accepted treatment protocol in recurrent poor responders. The current treatment strategies in poor responders include higher doses of gonadotropins ( over 450-600 IU/ day ) (1), use of antagonists (2,4), microdose flare (4,6) and growth hormone (7,8). Adjuvant therapies such as dehydroepiandrosterone ( DHEA ) (9), oral contraceptive pills, progestins (10), steroids (11), L-arginine (12) and low dose aspirin (13) have also been used in order to improve ovarian response and pregnancy rates in poor responders. Modifying controlled ovarian hyperstimulation ( COH ) with clomiphene citrate ( CC ) or letrozole ( L ) in addition to gonadotropins is promising and has gained acceptance for use in these cases (14,17). CC binds hypothalamic estrogen receptors and induces gonadotropin releasing hormone ( GnRH ) secretion by altering the negative feedback effect of estrogen on the hypothalamus. Triggered GnRH secretion increases pituitary gonadotropin release and finally results in stimulated ovarian follicular activity. The main benefits of adjunctive use of aromatase inhibitors ( AI ) in cycles of poor responders were reduced costs and cycle cancellation rates with comparable pregnancy outcomes (18,19). However, in the literature, there is one report that compares the effectiveness of CC and AI in poor responders in intracytoplasmic sperm injection ( ICSI ) cycles (16) and yet there is no study comparing these agents in recurrent poor responders. 

In this study, we attempted to clarify the effectiveness of CC or L adjunctive to antagonist cycles stimulated with human menopausal gonadotropin ( hMG ) in poor prognosis IVF women who failed previous cycles with microdose or antagonist protocols. 

## Materials and Methods

### Cases

One thousand and one hundred IVF cycles at Gazi University School of Medicine-based infertility clinic, Ankara, Turkey, from January 2006 to December 2009 were reviewed and 42 cycles of 32 infertile women who underwent IVF with at least 2 cycles of microdose flare or GnRH antagonist protocol and who failed to have ideal follicles to be retrieved during ovum pick-up ( OPU ) as a result of poor response to gonadotropin stimulation were retrospectively evaluated in this study. The Institutional Review Board and Ethics Committee of Gazi University School of Medicine approved this retrospective cohort study. 

## Ovarian stimulation protocols

**Fig.1:**
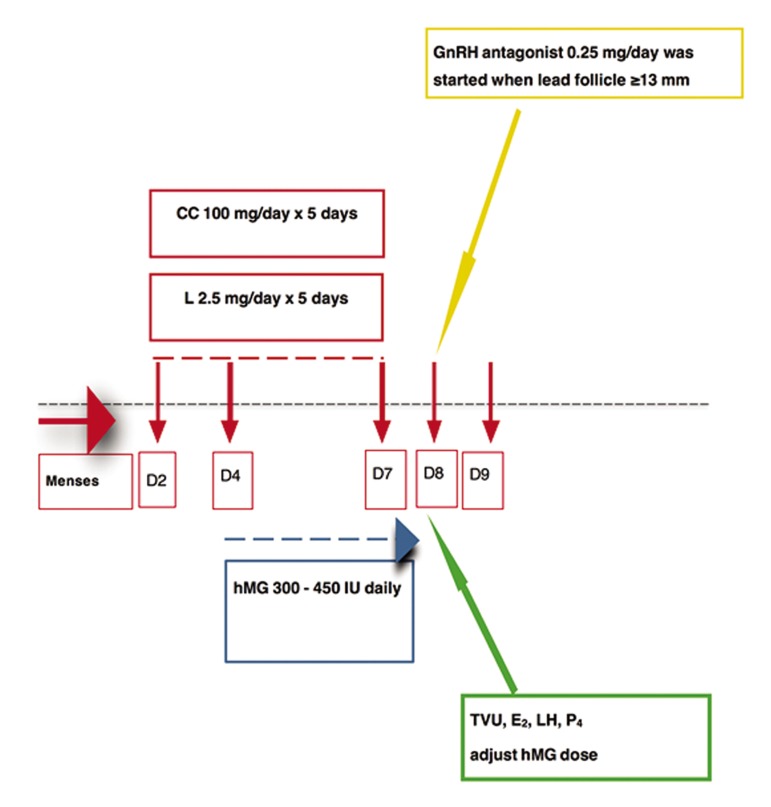
Schematic representation of CC vs. L+hMG+antagonist protocols. CC; Clomiphene citrate, L; Letrozole, hMG; Human menopausal gonadotropin, GnRH; Gonadotropin releasing hormone, TVU; Transvaginal ultrasound, LH; Luteinizing hormone, D; Day, E_2_; Estradiol, and P_4_; Progesterone.

Women (n=32) were equally divided into two groups, as CC and L groups, based on receiving CC (Serophene^®^, Serono, Turkey) 100 mg/day and L (Femara^®^, Novartis, Turkey) 2.5 mg/day, beginning on day 2 of the cycle and continued for 5 days. On day 4 of the cycles, hMG (Merional ^®^, IBSA, Turkey ) 300-450 IU/d administration was initiated. Daily GnRH antagonist (0.25 mg of cetrorelix acetate, Cetrotide^®^, Serono, Turkey) was started when the leading follicle exceeded ≥13 mm in diameter and continued until the day of human chorionic gonadotropin (hCG) administration. Recombinant hCG (250 mcg prefilled syringe, Ovitrelle^®^, Merck Serono, Turkey) was administered subcutaneously (SC) for final oocyte maturation when two or more leading follicles were ≥ 17 mm in diameter. The endometrial thickness was also documented via transvaginal ultrasonography (TVU) on the day of hCG administration. Schematic representation of the CC/L+hMG+antagonist protocols was shown in figure 1.

## Oocyte retrieval, embryo transfer and luteal support

Oocyte retrieval was performed under TVU guidance 35-36 hours after hCG administration and all women had intravenous sedation with midazolam (Dormicum^®^, Roche, Turkey). Metaphase II (M2) oocytes were fertilized with ICSI instead of conventional IVF to minimize the risk of fertilization failure. Depending on the women’s age, quality and number of available embryos, 1-4 embryo transfer (ET) was performed under TVU guidance 48-72 hours after OPU. Luteal phase was supported with 90 mg intravaginal progesterone gel (Crinone 8% gel^®^, Merck Serono, Turkey). 

## Detection of pregnancy

Pregnancy testing was performed by determining the quantitative serum hCG level at 12 days after ET, while intrauterine pregnancy was confirmed using TVU 2 weeks after a positive pregnancy test. A clinical pregnancy was defined as a positive serum beta hCG ( βhCG ) test result with the presence of a gestational sac on TVU or by histologic examination of products of conception in women who were aborted. 

## Outcome measures and statistical analysis

Main outcome measures were overall pregnancy, clinical pregnancy and live birth rates per cycle. The duration of stimulation, mean gonadotropin dose used, endometrial thickness, number of mature follicles, serum estradiol (E_2_) and progesterone (P) levels on the day of hCG administration, the number of retrieved oocytes and fertilization rates were also evaluated. The statistical analysis was performed using the Statistics Package for Social Sciences version 12.0 (SPSS, SPSS Inc., Chicago). The Chi square (χ2) test and Fisher’s exact test were used to analyze nominal variables in the form of frequency tables. Normally distributed (Kolmogorov-Smirnov test) parametric variables were tested by independent Student’s t test. Nonnormally distributed metric variables were analyzed by Mann-Whitney U test. A value of P<0.05 was considered statistically significant. Values were expressed as mean ± standard deviation (SD) unless otherwise stated.

## Results

A total number of 42 cycles of 32 severe poor responders were evaluated in this study. There were 22 cycles of 16 cases in the CC group and 20 cycles of 16 cases in the L group. The baseline characteristics of both groups were given in table 1. The overall cancellation rate was 78.5% and the pregnancy rate per attempted cycle was 7.1%. 

The women in both CC and L protocol groups were comparable regarding age (37.7 ± 6 vs. 36.3 ± 4.2, respectively), basal FSH level (13.3 ± 4.9 vs. 14.6 ± 4, respectively) and antral follicle count (2.1 ± 1.1 vs. 2.1 ± 1.1, respectively). Mean total dose of FSH used was significantly lower (1491 ± 873 vs. 2808 ± 1581.1 IU, P=0.005) and mean E_2_ level on the day of hCG injection was significantly higher (443.3 ± 255.2 vs. 255.4 ± 285.2 pg/mL, P=0.03) in the CC when compared to the L group. Other cycle characteristics and cancellation rates were similar in both groups. However, the ET rate was significantly higher in CC protocol (27.2%) when compared to that of the L protocol (15%, P<0.05, Table 1).

The overall pregnancy and live birth rates per attempted cycles were significantly higher in CC protocol than L protocol ( 13.6 vs. 0% and 4.5 vs. 0%, respectively, P<0.05, Table 1). 

**Table 1:** Comparison of baseline characteristics, COH response and pregnancy outcomes between CC and L+ GnRH antagonist protocols


Variable	CC n=16	L n=16	P value

No. of cycles	22	20	0.16
Female age (Y)	37.7 ± 6	36.3 ± 4.2	0.07
Day 3 serum FSH (mIU/mL)	13.3 ± 4.9	14.6 ± 4.2	0.56
Antral follicle count	2.1 ± 1.1	2 ± 1.2	0.32
Duration of stimulation (days)	12 ± 3.4	11.6 ± 2.8	0.43
Total dose of FSH used (IU)	1491 ± 873	2808 ± 1581.1	0.005
E_2_ level on the day of hCG injection (pg/mL)	443.3 ± 255.2	255.4 ± 285.2	0.03
P level on the day of hCG injection (ng/mL)	0.6 ± 0.7	0.9 ± 1.1	0.29
Endometrial thickness on the day of hCG administration (mm)	9.1 ± 2.4	8.6 ± 3.7	0.07
Follicles ≥ 17 mm on hCG (day)	1.1 ± 0.7	1.1 ± 0.7	0.96
Follicles 12-16 mm on hCG (day)	1.8 ± 1.5	1.6 ± 1.5	0.91
No. of canceled cycles, %	72.7	85	0.1
No. of canceled cycles due to poor ovarian response, %	63.6	70	0.2
No. of oocyte-cumulus complexes	2.5 ± 1.4	3.3 ± 1.3	0.52
No. of metaphase II oocytes	2.0 ± 1.4	2.6 ± 1.7	0.83
M2/no. of oocyte-cumulus complexes, %	80	80	0.59
Fertilization rate, %	70.7	80	0.65
ET rate, %	27.2	15	0.04
No. of ET	1.6 ± 0.8	2.3 ± 1.1	0.42
No. of ET with less than 10% fragmentation and blastomere number ≥ 7	0.8 ± 1.1	1.0 ± 0.1	0.09
Pregnancy rate per cycle attempt, %	13.6	0	<0.05
Pregnancy/ET, %	50	0	<0.05
Biochemical pregnancy rate per cycle attempt, %	4.5	0	<0.05
Biochemical pregnancy/ ET, %	16.6	0	<0.05
Clinical pregnancy rate per cycle attempt, %	9	0	<0.05
Clinical pregnancy/ET, %	33.3	0	<0.05
Miscarriage rate, %	33.3	0	<0.05
Live birth rate per cycle attempt, %	4.5	0	<0.05
Live birth/ET, %	16.6	0	<0.05


Data presented as mean ± standard error (SE). CC; Clomiphene citrate, L; Letrozole, ET; Embryo transfer, COH; Controlled ovarian hyperstimulation, GnRH; Gonadotropin releasing hormone, FSH; Follicle stimulating hormone, hCG; Human chorionic gonadotropin and M2; Metaphase II.

## Discussion

We used CC and L in cases of IVF with previous attempts resulting with cancelation due to poor response to gonadotropin stimulation and an ovum pick up was not completed under either flare or antagonist protocol. Although the definition of "severe poor responder" did not exist in the literature, we used this term to indicate very poor prognostic cases before an adoption or oocyte donation were advised to the couples. Our study revealed that the adjunctive use of CC is more effective in reducing hMG dose, increasing the number of embryos transferred and achieving better pregnancy rates than AI in severe poor responders. Both groups were comparable in the number of retrieved oocytes and cancellation rates. Unlike previous reports regarding adjunctive use of CC or L in poor responders, our higher cancellation rates ( 78.5% ) might be attributed to allocation of more severe, recurrent poor prognostic cases into our study. 

Microdose flare and GnRH antagoists are mostly accepted as first line protocols in poor responders (20). The adjunctive use of AI or CC may be helpful in their subsequent ICSI cycles. There is little but encouraging evidence for using these agents in poor responders (16,21). In a subgroup analysis of a study performed by Jovanovic et al. (16) there were comparable improvements in COH response and cycle cancellation rates ( 39.8 ± 8.5% vs. 24.8 ± 7.6%, respectively ) with the adjunctive use of CC vs. L plus high dose gonadotropins in 29 poor responders, only 2 clinical pregnancies and one live birth were reported in group L but none in group CC. 

Regarding our data, it should be stated that the adjunctive use of L has little advantage in improving pregnancy outcomes in severe poor responder women. In the current study, we observed that adjunctive use of L failed to increase pregnancy rates despite its useful effects on ovarian response. L increases local androgen levels in the follicle and this hyperandrogenic environment in the follicle might impair oocyte quality and be responsible for poor pregnancy outcome (18,22,23). However, different outcomes in terms of quantity of the oocytes retrieved, quality of the embryos and pregnancy success concerning the use of L were previously reported (16,18,24,27). CC stimulates ovarian follicle development and maturation by inducing endogen gonadotropin secretion and aromatase activity, indirectly (28). The opposite effects of CC and L on aromatase enzyme activity may be the main cause of different pregnancy outcomes. AI treatment as an adjunctive therapy has been administered at a standard dose for a standard duration. It is possible that different infertile women with different aromatase activities require an individualized dosage in order to attain the desired effect and maximize the benefit of AI. 

It must be noted that the retrospective design and low number of cycles weakened the power of our results. The burden of financial costs and the psychological aspect of recurrent failure lead to a high drop-out rate in these couples (29). For this reason, it is difficult to find high number of severe poor responder cases and perform a more powerful prospective randomized study. Therefore, most previous similar analyses in the literature were also in retrospective design with low number of cycles (16,17). In another retrospective study, Yarali et al. (15) compared the effectiveness of L/antagonist protocol with microdose flare in 885 poor responder women and concluded that L plus antagonist has similar efficiency in terms of cycle characteristics and pregnancy outcome. However, the women had more than 4 M2 oocytes in each group, which indicates a population with more favorable prognosis as compared to our population. In fact, bias cannot be eliminated without randomization as a nature of retrospective studies (30). However, in a recent randomized study L/antagonist protocol was found better than microdose flare up in decreasing the days of stimulation and doses of used gonadotropin in poor responders’ ICSI cycles (31). 

CC significantly improves COH response by decreasing the doses of used gonadotropin and duration of stimulation without altering endometrial development in gonadotropin plus antagonist protocols in poor responders (32). Although pregnancy rates of adjunctive use of CC to gonadotropin were comparable with microdose flare up or antagonist protocols in poor responders, addition of CC seems to be beneficial for reducing costs (32,33). In a recent report from a group of women with severe poor response to gonadotropin stimulation, high doses of gonadotropins were used on the subsequent cycle and clinical pregnancy rate was 5.6% with a mean costs per cycle and per live birth of €5597 and €124,540, respectively (29). In that analysis, some women preferred a milder stimulation with CC and authors concluded that all results were similar with CC as compared to gonadotropins. 

## Conclusion

Severe poor responders who had previously failed to respond to microdose flare protocol or GnRH antagonist protocol may benefit from CC+GnRH antagonist protocols despite a high cancellation rate. CC+GnRH antagonist protocols may provide an alternative option for severe poor responders with low costs. Further prospective randomized studies are needed to confirm these results or to determine better one in severe poor responder women. 
